# Smart grid data compression and reconstruction by wavelet packet transform

**DOI:** 10.1016/j.mex.2024.102872

**Published:** 2024-07-20

**Authors:** Rakhi Jadhav, Anurag Mahajan

**Affiliations:** Electronics and Telecommunication Engineering, Symbiosis Institute of Technology, Symbiosis International (Deemed University), Pune 412115, India

**Keywords:** Wavelet Packet Transform, Discrete wavelet transform, Reconstruction error, Compression ratio

## Abstract

A smart grid is a power network from generation to consumers and provides beneficial, steady, and safe electricity. It utilizes smart meters for billing, phasor measurement units to check the system's health., etc. As a result, it contains enormous volumes of real-time data that may be shared and stored by users, control centers, and services in a smart grid. It weakens the smart grid's communication networks. The size of the data in a smart grid will grow extremely in the future. As a result, it must reduce distortion in data compression and denoise while minimizing the demand on storage and communication networks. The goal of data compression and denoising should be to maximally conserve the useful data while accurately reflecting the state of the system and providing sufficient data regeneration at the receiving end. This paper has used lower-order different wavelets to represent a design to compress and reconstruct data at level three using wavelet Packet Transform. It works on the phasor measurement unit's current magnitude and voltage sag signals.

• The proposed design has a better compression ratio.
• Low reconstruction error.
• This design is easy to access, systematic, profitable, and not time-consuming.

## Highlights

• The proposed design has a better compression ratio.
• Low reconstruction error.
• This design is easy to access, systematic, profitable, and not time-consuming.

## Graphical abstract

**Figure:**
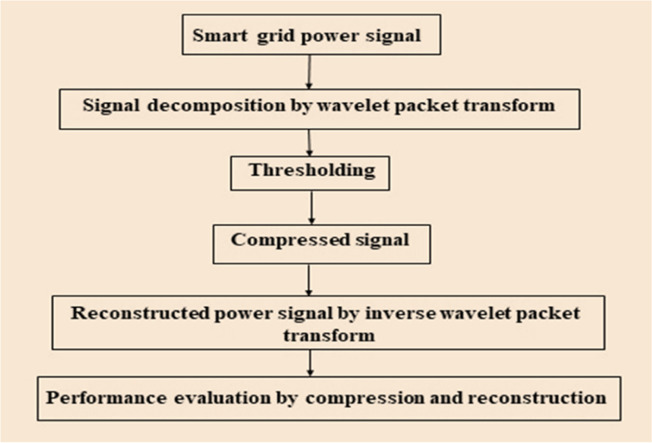

Specifications table

**Table:** 

Subject area:	Engineering
More specific subject area:	Data compression and reconstruction
Name of your method:	Wavelet Packet Transform
Name and reference of original method:	Wavelet Packet Decomposition
	J. Khan, S. Bhuiyan, G. Murphy, and J. Williams, Data Denoising and Compression for Smart Grid Communication, IEEE Trans. Signal Inf. Process. over Networks. 2(2016) 200–214. https://doi.org/10.1109/TSIPN.2016.2539680
Resource availability	MATLAB 2021a

## Method details

The energy requirement is increasing due to the rise in population. Conventional sources supply most of the demand, constantly reducing and causing severe environmental problems. The existing power grid is unpredictable, inferior, and provides insufficient electricity. The smart Grid is an improvement growing with various technologies [1]. The distribution network will install many smart monitors and meters to allow comprehensive and actual data observation. It requires computations and communication to process and share details. Hence it needs low data storage and adequate bandwidth for transmission. There are lossless and lossy techniques used in compression. It distorts the reconstructed signal in the lossy compression method. The Wavelet transforms under lossy methods are well with time and frequency localization. It is renowned for compressing variable frequency signals, non-periodic and transients, and describing the data with few coefficients [[2], [3], [4]]. The electrical signal disturbances are voltage fluctuations, sag, swell, harmonics, impulse, etc., employing wavelet transform to segment [5]. Wavelet Transform and the flexible threshold separate the disturbances like variable voltage, transients with impulse and oscillations, and distortions in the harmonic[[6], [7]]. Phasor Measurement Unit (PMU) signal compression using wavelet transform with Daubechies 2 is better than Coiflet 1 wavelet [8]. Discrete wavelet transform(DWT) and wavelet packet transform (WPT) compress and reconstruct the data by threshold. The minimum description length (MDL) decides the suitable wavelet and most coefficients for signal reconstruction [[9], [10]]. Compressing and reconstructing actual and simulated oscillations decide a suitable scale and wavelet for decomposing data [11]. Wavelet Transform effectively compresses the voltage sag signal in the smart grid [12]. It compresses and analyzes data using wavelet transform [[13], [14], [15], [16]].

It compresses and denoises smart grid system data by embedded zero tree wavelet transform(EZWT) [[17], [18], [19]]. Wavelet packet decomposition (WPD) analyses, compresses, denoises, and reconstructs data by calculating a level-wise threshold [20]. WPT compresses and reduces noise in the smart grid data. A threshold operates on the basis decided by the modified Shannon entropy i.e. weighted Entropy [[21], [22]]. Different wavelets are applied to reduce data storage and complexity using discrete wavelet transform [23]. There can be better compression and reconstruction with a reduction of noise and complexity for smart grid signals using wavelet transform [24]. It operates different wavelets, improves compression, and reduces noise by wavelet packet transform [25]. It decides the wavelet function and the appropriate decomposition level for high compression of power quality disturbance signals [26].

The compressed sensing (CS) method detects frequencies causing harmonic distortion in voltage and current signals. It measures a matrix from a few random samples. It compresses the signal by converting the time domain signal to the frequency domain using a linear transformation. Then it reconstructs the signal with a few samples of the power signal by the inverse linear transformation [27]. It researches regenerated signals after supply failures, pursuing the compressed sensing methods of basis, matching, and orthogonal matching search. It uses a heuristic, i.e., brute force, to get the quasi-optimal number of atoms in the instantaneous current and voltage signals. The minimum number of samples reconstruct the original signal from inadequate and random samples. The matching pursuit (MP) takes the least time and more samples than orthogonal matching pursuit (OMP). Basis pursuit (BP) and orthogonal matching need a long time to process a few scanty and arbitrary samples to recover the signal [28]. In the smart grid, compressed sensing reconstructs and examines the wireless sensor networks' power quality. Up to 50 % of Nyquist's, voltage and current data from smart grids are evaluated and sampled using the Similarity and Threshold Regularized Orthogonal Matching Pursuit (STROMP) method [30].

The wavelet transform with multiresolution analysis (MRA) uses Daubechies wavelet of order two at decomposition level five to compress and denoise power system signals in Smart Grid [29]. Electrical disturbance signal is compressed by wavelet packet-enhanced arithmetic coding, which divides the signal into high and low frequency components for the optimal basis [31]. Wavelets are appropriate for transient and discontinuous signals since they offer basis functions with a time-frequency. Wavelets are basic, fixed structures that express signals at the right levels and locations. Disturbance signals are compressed by the wavelet packet and discrete wavelet transforms. It displays a Wavelet Transform (WT) and Discrete Cosine Transform (DCT)-based comparative compression of an actual disturbance from a digital fault recorder. It effectively retrieves data about faults and disturbances from remote fault recorders and relays. Better CR and SNR necessitate appropriate selection of WT's order and scale [32]. Because of the segments in the power quality meters in the Smart Grids, determining the beginning and end of disruptions is difficult. Wavelet transform is used by electrocardiograms to compress large amounts of data by breaking it down, applying threshold, encoding it with various wavelets, and then reconstructing it. The signal is more effectively compressed by the HAAR wavelet and local threshold [33].

Fuzzy transform is used in smart grids to minimize electrical signals. This creates and applies a matrix-based fuzzy transform for 2-D smart grid data compression using the least squares (LS) approximation. The 2-D data reduction matrix is created by adding up the 1-D power signals. From the data matrix division, create sub-matrices, and then compress each one using the discrete F transform with fuzzy partitions. In the LS approximation, the unknown variables are substituted for the discrete F-transform's variables, and the discrete inverse F-transform is generated by reducing the variation with respect to these unknown factors [22,34].

The enhanced disturbance compression method (EDCM) for voltage and current signals is based on the fundamental, harmonic, and transient coding method (FHTCM). It determines the amplitude, frequency, and phase of the fundamental and harmonic parts using notch filtering—warped discrete Fourier transform. It uses the minimal description length wavelet transform to compress only the transient coefficients. FHTCM outperforms WT-based and EDCM compression techniques [35]. In order to estimate the fundamental sinusoidal component, it suggests an enhanced data compression method, which outperforms the standard disturbance compression method (SDCM) in terms of CR and SNR [36].

The approach in [19,23,[25], [26]] and [28] is computationally complex, decomposing at level three with EZWT including a more significant number of higher-order filters, biorthogonal wavelets (bior4.4) in [19]. Smart grid data is compressed and denoised using wavelets db3 at level1, db2 at levels 2 and 3, and db1 at level 4 by DWT [23]. It reduces and denoises smart grid data with db3 at level1, db2 at levels 2, 3 and 4, and db1 at level 5 with WPT [25]. It compresses voltage sag signal using a db4 wavelet at level 3 by wavelet transform [26]. The compressed sensing method is complex due to matrix [28].

It is possible to reduce the complexity further and improve the compression ratio and reconstruction error. Therefore, the new method utilizes wavelet packet transform, integrating various low-order wavelets with a level wise appropriate threshold. The designed process will reduce complexity with better compression and minimum distortion at level 3 of decomposition. It compares the results with [19,23,[25], [26]] and [28].

The present design depends on wavelet packet transform to compress and reconstruct data in a smart grid. The discrete wavelet transform develops into a wavelet packet tree DWT transmits a signal across a low-pass filter (LPF) and a high-pass filter (HPF) and down samples it by two employing the Pyramid Algorithm. The detail coefficients contain noise due to their high frequency; hence they are applied to threshold. It regenerates a signal by inverse discrete wavelet transform (IDWT). WPT represents the signal better with a proper subtree [10,12]. It applies a threshold to the wavelet coefficients to compress and reduce noise and regenerate the signal. A hard threshold equals the component zero, having absolute values lower than a threshold. Soft-threshold equals zero; the components having absolute values lower than a threshold then reduce the non-zero components to zero [20].

The compression maintains the coefficients with disturbances and neglects the coefficients free from disturbances. It distorts the data due to the suppression of the noise. It applies the threshold $t_{k}^{m}$ to the absolute maximum value of the original detail coefficients-$C_{k}^{m}$, as displayed below at the kth node of level m.

(1) $$t_{k}^{m}=\left(1-u\right)xmax\;\left|C_{k}^{m}\right|$$

It selects 0≤u≤1. If u =0.1 then threshold is 0.9 i.e. 90 % in Eq. (1). It compresses the data better as it applies threshold to detail coefficients, but the reconstruction error is significant. The hard threshold operates as shown below [12] in Eq. (2).

(2) $$C_{k}^{mm}=\left\{ \begin{array}{c} C_{k}^{m}\;\;\;\;\;\;\;\;\left|C_{k}^{m}\right|\geq \;t_{k}^{m}\\ 0\;\;\;\;\;\;\;\;\;\;\;\;|C_{k}^{m}|<\;t_{k}^{m} \end{array}\right.$$

The soft threshold performs as shown below [20] in Eq. (3).

(3) $$C_{k}^{mm}\;=\{\begin{array}{c} sign\left(C_{k}^{m}\right)\left(\right|C_{k}^{m}|-\;t_{k}^{m});\;\;\;\;\;\text{if}\left|C_{k}^{m}\right|>t_{k}^{m}\\ 0\;;\;\;\;\;\;\;\;\;\;\;\;\;\;\;\text{if}\;|C_{k}^{m}|\leq t_{k}^{m} \end{array}\;$$

The coefficients $C_{k}^{\text{mm}}$ can have soft or hard-threshold.

**Fig. 1:**
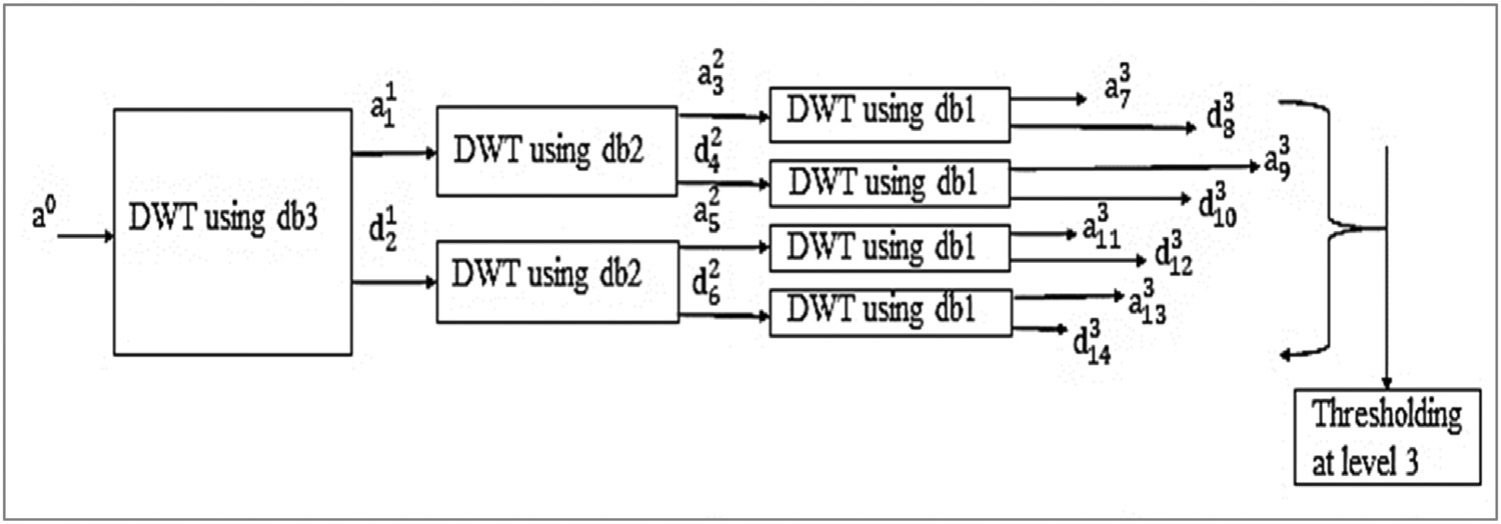
Decomposition by Proposed design using wavelet packet transform.

**Fig. 2:**
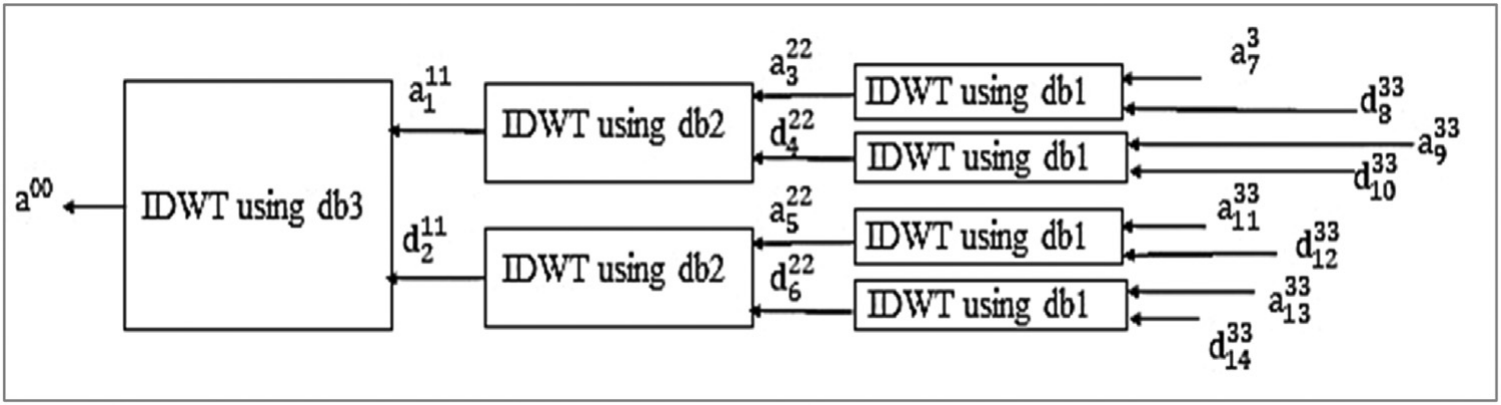
Reconstruction by Proposed design using wavelet packet transform.

In Fig. 1, DWT further decomposes approximations and detail coefficients to obtain the complete wavelet packet tree. In the compression, it selects Daubechies filters db3 at level 1, db2 at level 2, and db1 at level 3, and for reconstruction, db1 at level 3, db2 at level 2, and db3 at level 1, as indicated in Fig. 2. It decomposes the signal $a^{0}$ using a db3 wavelet at level 1 by DWT into approximation coefficients $a_{1}^{1}$ and detail coefficients $d_{2}^{1}$. It further disintegrates the approximation $a_{1}^{1}$ into approximation coefficients $a_{3}^{2}$ and detail coefficients $d_{4}^{2}$ using db2 wavelet by DWT at level 2. The detail coefficients $d_{2}^{1}\;$decomposes from level 1 into approximation $a_{5}^{2}$ and detail coefficients $d_{6}^{2}$ using the db2 wavelet by DWT at level 2. It further decays the approximation coefficients $a_{3}^{2}$ into $a_{7}^{3}$ and $d_{8}^{3}$, and detail coefficients $d_{4}^{2}$ into $a_{9}^{3}$ and $d_{10}^{3}$ from level 2 using db1 wavelets by DWT at level 3. Similarly, $a_{5}^{2}$ and $d_{6}^{2}$ are decomposed further from level 2 to level 3 by DWT using db1.

The optimal tree determines the coefficients for the compression and then reconstructs the signal by inverse discrete wavelet transform from the compressed signal. In the compression, the coefficients $a_{7}^{3}$ are pure approximation coefficients, and therefore it does not apply threshold to them. The detail coefficients $d_{8}^{3}$, $a_{9}^{3}$, and $d_{10}^{3},{\;\;a}_{11}^{3}$, $d_{12}^{3}$,${\;a}_{13}^{3}$, and $d_{14}^{3}\;$ are applied to hard threshold using Eq. (2) into coefficients $d_{8}^{33}$, $a_{9}^{33}$ and $d_{10}^{33}$ and ${\;a}_{11}^{33}$, $d_{12}^{33}$, $a_{13}^{33}$, and $d_{14}^{33}\;$ respectively. The coefficient $a_{3}^{22}$ is reconstructed by IDWT using db1 for the coefficients $a_{7}^{3}$ and $d_{8}^{33}$ at level 3. The coefficient $d_{4}^{22}$ is recreated by IDWT using db1 for the coefficients $a_{9}^{33}$ and $d_{10}^{33}$ at level 3. Similarly, the coefficients $a_{5}^{22}$ and $d_{6}^{22}$ are regenerated at level three by IDWT using db1 wavelet. The coefficient $a_{1}^{11}$ is reconstructed by IDWT using db2 for the coefficients $a_{3}^{22}$ and $d_{4}^{22}$ at level 2. The coefficient $d_{2}^{11}$ is recreated by IDWT using db2 for the coefficients $a_{5}^{22}$ and $d_{6}^{22}$ at level 2. Finally, the coefficient $a^{00}$ is regenerated by IDWT using db3 for the coefficients $a_{1}^{11}$ and $d_{2}^{11}$ at level 1. The non-zero coefficients are the summation of pure approximation coefficients at level 3, $a_{7}^{3}$, other coefficients $d_{8}^{33}$, $a_{9}^{33}$ and $d_{10}^{33}$ and ${\;a}_{11}^{33}$, $d_{12}^{33},{\;a}_{13}^{33}$, and $d_{14}^{33}\;$at level 3 after applying threshold.

## Method validation

### Datasets

To create the simulated faulty data of PMU current and voltage sag signals, MATLAB 2021a SIMULINK is utilized. A three phase to ground fault provides PMU current. To assess the proposed design in Table 1, 2000 samples of the PMU fault current are taken. Furthermore, in order to run the suggested design in Table 2, the voltage sag signal is investigated for 2816 samples. For comparison in Table 3, different numbers of 357, 379,412, 501, and 512 samples are created for the voltage sag signal.

**Table 1:** Comparison of proposed design for PMU current signal.

	Method	Original Samples	Wavelets and Decomposition Level	% CR	NRMSE 2
Proposed design	WPT	2000	db3, db2, db1, Level 3	16.15	1.2x$10^{-3}$
[19]	EZWT	2000	bior4.4, Level 3	35.06	3.88x x$10^{-3}$
[23]	DWT	2000	db3, db2, db2, db1 Level 4	30.40	0.14
[25]	WPT	2000	db3, db2, db2, db2, db1 Level 5	30.25	0.10

**Table 2:** Comparison of proposed design for Voltage sag signal.

	Method		Original Samples	Wavelets and Decomposition Level	%CR	NMSE
Proposed design	WPT		2816	db3,db2,db1,Level 3	25.46	5.14x$10^{-5}$
[26]	DWT		2816	db4, Level 3	27.10	5.25x$10^{-5}$
[25]	WPT		2816	db3, db2, db2, db2, db1, Level 5	25.96	4.60x$10^{-3}$

**Table 3:** Comparison of proposed design for Voltage sag signal.

	Method	Original **Samples**	Level of Decomposition	% CR	% Reconstruction	NRMSE 1
**Proposed design**	WPT	412	Three	19.66	99.97	4.69x$10^{-4}$
[28]	CS(MP)	412	–	–	90.89	–
[25]	WPT	412	Five	19.66	99.91	5.81x$10^{-4}$
**Proposed design**	WPT	357	Three	19.33	99.97	5.20x$10^{-4}$
[28]	CS(OMP)	357	–	–	90.19	–
[25]	WPT	357	Five	19.33	99.91	6.25x$10^{-4}$
**Proposed design**	WPT	379	Three	19.00	99.98	4.98x$10^{-4}$
[28]	CS(OMP)	379	–	–	97.46	–
[25]	WPT	379	Five	19.00	99.79	6.06x$10^{-4}$
**Proposed design**	WPT	501	Three	18.76	99.90	3.69x$10^{-4}$
[28]	CS(BP)	501	–	–	97.57	–
[25]	WPT	501	Five	18.76	99.33	5.31x$10^{-4}$
**Proposed design**	WPT	512	Three	19.53	99.89	2.86x$10^{-4}$
[28]	CS(BP)	512	–	–	98.20	–
[25]	WPT	512	Five	19.53	99.67	4.78x $10^{-4}$

**Fig. 3:**
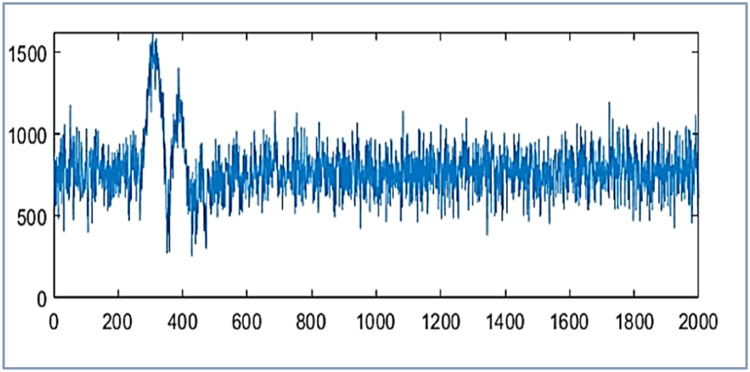
PMU current magnitude original signal.

**Fig. 4:**
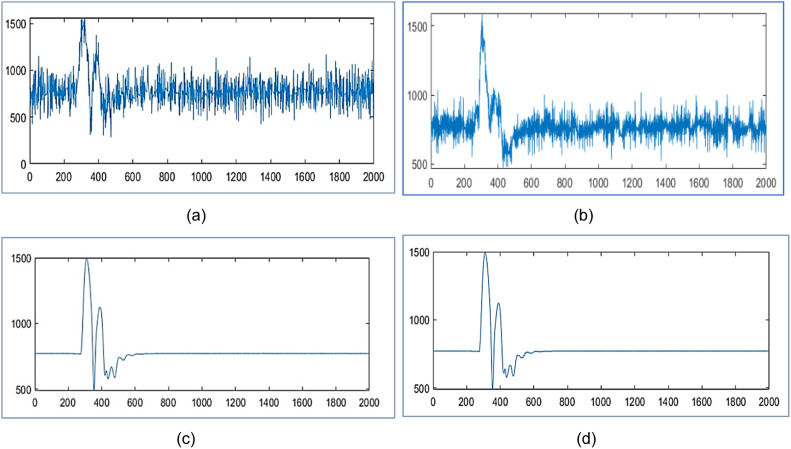
PMU current magnitude signal (a) Reconstructed signal for algorithm in [23] with CR 30.40 %, NRMSE 2 of 0.14 (b) Reconstructed signal in [25] with CR 30.25 % and NRMSE 2 of 0.10 (c) Reconstruted signal from proposed design using WPT with CR 28.90 %, NRMSE 2 of 7.36x$10^{-4}$ (d) Reconstruted signal from proposed design using WPT with CR 16.15 %, NRMSE 2 of 1.2x$10^{-3}$.

**Fig. 5:**
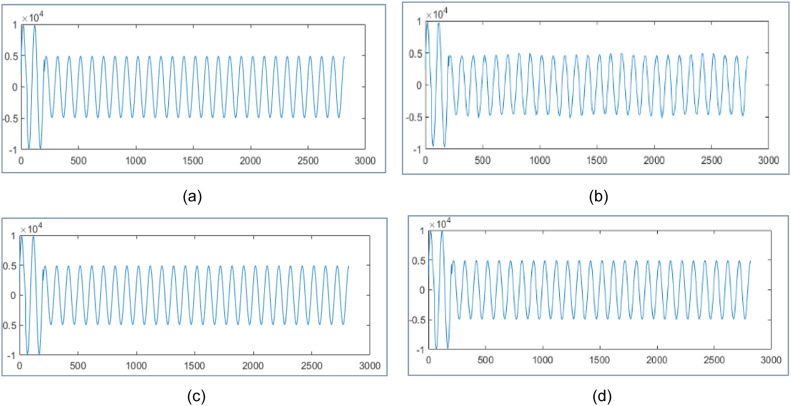
Voltage sag signal (a) Original signal and (b) reconstructed signal for algorithm in [25] with CR 25.96 % and NMSE 4.60x$10^{-3}$ (c) Reconstruted signal from proposed design using WPT with CR 26.17 %, NMSE 3.82x $10^{-5}$ (d) Reconstruted signal from proposed design using WPT with CR 25.46 %, NMSE 5.14x $10^{-5}$.

The proposed design tests wavelet packet transform on MATLAB 2021a algorithm for data compression and reconstruction. It operates two thousand samples of simulated PMU current magnitude signal [19] and compares the results with [19,23,25] as shown in Fig. 3, Fig. 4 and Table 1. It also operates 2816 samples of simulated voltage sag signal [26] and compares the results with [[25], [26]] as shown in Fig. 5 and Table 2. It also tests different number of samples of simulated voltage sag signal and compares the results with [25] and [28] in Table 3.

The performance parameters of proposed design are% compression ratio(%CR) and the reconstruction error as normalized root mean square error (NRMSE) as in [[19], [20]]. The performance of proposed design is also tested for% compression ratio(CR), and reconstruction error as normalized mean squared error(NMSE) as in [26]. It calculates the absolute error between the original and the reconstructed signal. Then it obtains the percentage relative error e. It checks the performance of the present design by% reconstruction, as in [[27], [28]].

(4) $$\%\;\text{CR}=\frac{\text{No}\;\text{of}\;\text{non}\;\text{zero}\;\text{coefficients}\;}{\text{No}.\;\text{of}\;\text{coefficients}\;\text{in}\;\text{the}\;\text{original}\;\text{signal}}x100\%$$

(5) $$\text{NRMSE}\;1=\sqrt{\frac{\sum_{i=0}^{N-1}{\left[X\left(i\right)-X_{r}\left(i\right)\right]}^{2}}{N^{2}}}$$

(6) $$\text{NRMSE}\;2=\sqrt{\frac{\sum_{i=0}^{N-1}{\left[X\left(i\right)-X_{r}\left(i\right)\right]}^{2}}{\sum_{i=0}^{N-1}X{\left(i\right)}^{2}}}$$

(7) $$\text{NMSE}=\frac{\sum_{i=0}^{N-1}{\left[X\left(i\right)-X_{r}\left(i\right)\right]}^{2}}{\sum_{i=0}^{N-1}X{\left(i\right)}^{2}}$$

(8) $$e=\frac{\left|\sum_{i=0}^{N-1}\lceil x\left(i\right)-x_{r}\left(i\right)\rceil \right|}{x\left(i\right)}x100\%$$

(9) %Reconstruction=100−e

x(i), $x_{n}\left(i\right)$ and $x_{r}$(i) are the original, noisy and regenerated signals respectively, for i ranging from 0 to N-1 samples. NRMSE/NMSE decides how accurately the regenerated signal describes the changes in the original signal.

Fig. 3 represents the original PMU current signal of 2000 samples and its reconstructed signals are represented by Figs. 4(a) to 4(d). The original signal is noisy; hence it is applied to soft threshold by Eq. (3) for reducing noise by the appropriate percentage of the discrepancy between the noisy and the original signal. Then it is at hard threshold in Eq. (2) for compression. It suppresses the noise and almost reconstructs the quality signal.

In Fig. 4(a), it reconstructs the signal with CR 30.40 % and poor NRMSE 2 of 0.14 in [23] using DWT . In Fig. 4(b), it reconstructs the signal with CR 30.25 % and poor NRMSE 2 of 0.10 by WPT in [25]. In Fig. 4(c), it has reconstruction with CR 28.90 % and NRMSE 2 of 7.36x$10^{-4}$ by the proposed design with level three WPT. In Fig. 4(d), it has reconstructed with CR 16.15 % and NRMSE 2 of 1.2x$10^{-3}$ by the proposed design using WPT at level three. It compares the reconstructed signals by the proposed design in Table 1 with results in [19,23] and [25].

Table 1 observes that proposed design compresses and reconstructs better than [19,23,25] for the PMU current magnitude signal. The algorithm in [23] has better compression 30.40 % but a poor reconstruction error 0.14 compared to [19]. The algorithm in [25] has better compression 30.25 % but a poor reconstruction error 0.10 compared to [19]. The proposed design has compressed the PMU signal 16.15 % and achieved NRMSE 2 of 1.2x $10^{-3}$ which is still better than [19,23] and [25]. The proposed design is simple as it achieves the better results at level 3 using lower order wavelets as compared to [19,23] and [25].

Fig. 5 displays the original voltage sag signal of 2816 samples in Fig. 5(a). It is at hard threshold for compression and reconstruction as in Eq. (2). In Fig. 5(b), it describes the reconstructed signal with CR 25.96 % and NMSE 4.60x$10^{-3}$ using the algorithm [25]. In Fig. 5(c), it has reconstruction with CR 26.17 % and NMSE 3.82x$10^{-5}$ by proposed design using WPT. In Fig. 5(d), it reconstructs the signal with CR 25.46 % and NMSE 5.14x $10^{-5}$ by the proposed design using WPT. It compares the reconstructed signals by the proposed design in Table 2 with results in [[25], [26]].

Table 2 notices that the proposed design has better reduction and reconstruction than [[25], [26]] for the voltage sag signal. The proposed design has achieved compression ratio 25.46 % and NMSE 5.14x$10^{-5}$ which is better than [[25], [26]].

**Fig. 6:**
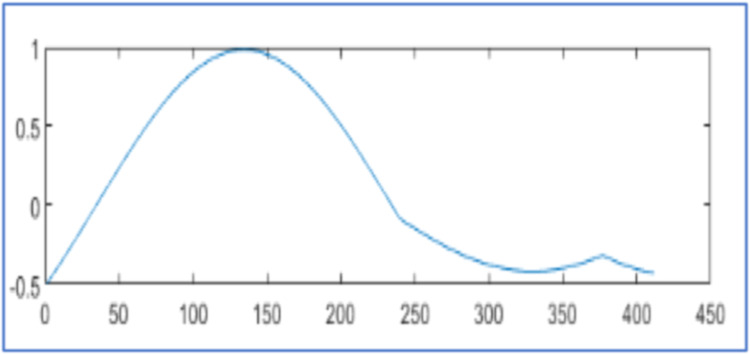
Voltage sag signal with 412 samples.

**Fig. 7:**
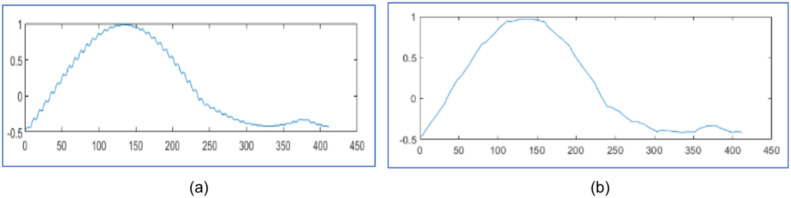
Voltage sag signal reconstruction for 412 samples (a) 99.97 % by proposed design using WPT with 81 samples (b) 99.91 % in [25] with 81 samples.

**Fig. 8:**
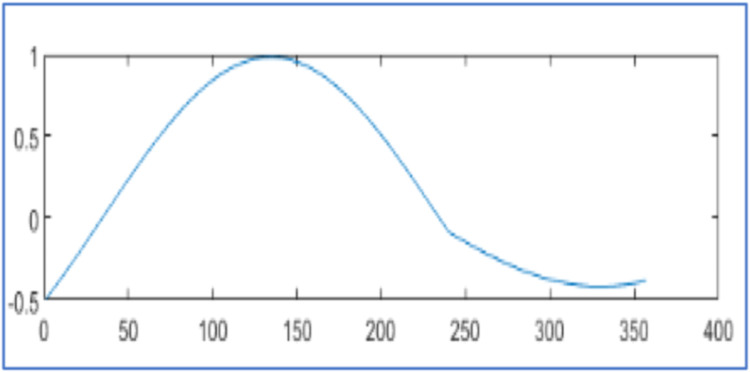
Voltage sag signal 357 samples.

**Fig. 9:**
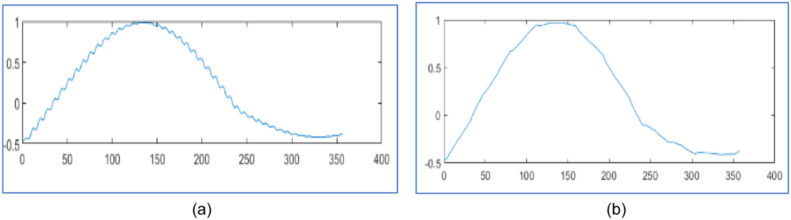
Voltage sag signal reconstruction for 357 samples (a) 99.97 % by proposed design using WPT for 69 samples (b) 99.91 % in [25] for 69 samples.

**Fig. 10:**
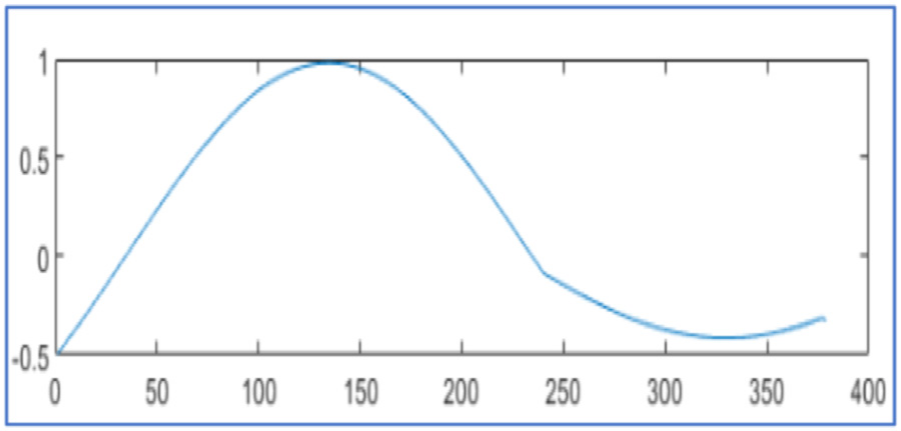
Voltage sag signal 379 samples.

**Fig. 11:**
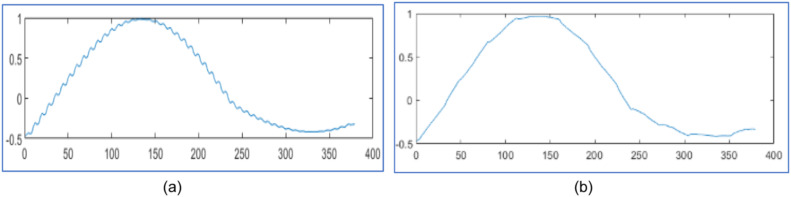
Voltage sag signal reconstruction for 379 samples (a) 99.98 % by proposed design using WPT for 72 samples (b) 99.79 % in [25] for 72 samples.

**Fig. 12:**
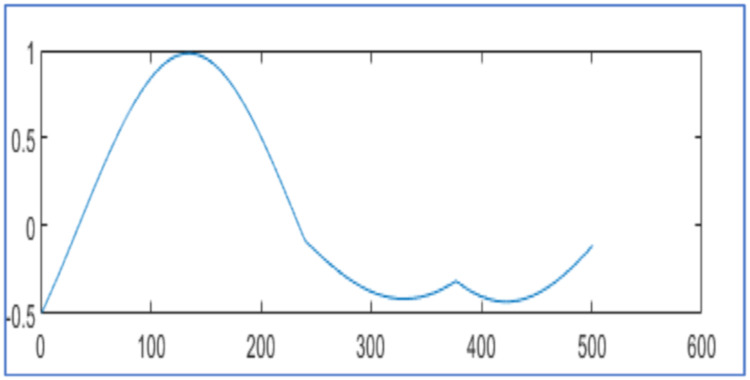
Voltage sag signal 501 samples.

**Fig. 13:**
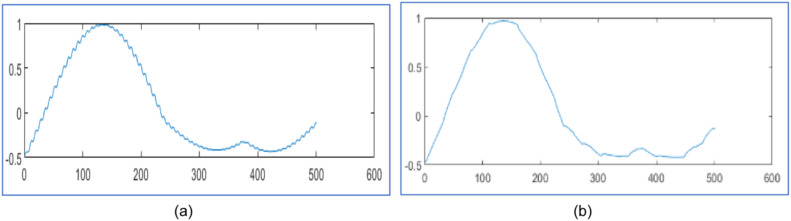
Voltage sag signal reconstruction for 501 samples (a) 99.90 % by proposed design using WPT for 94 samples (b) 99.33 % in [25] for 94 samples.

**Fig. 14:**
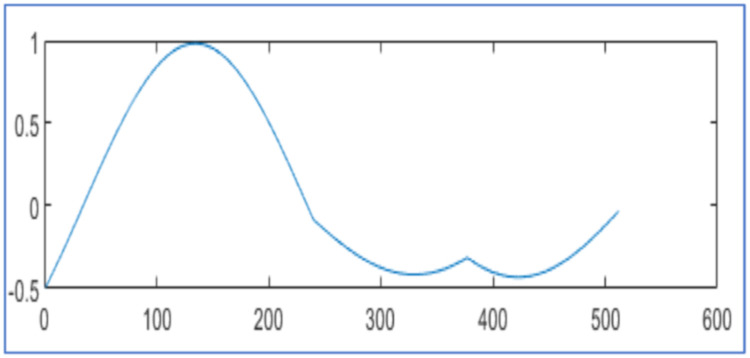
Voltage sag signal 512 samples.

**Fig. 15:**
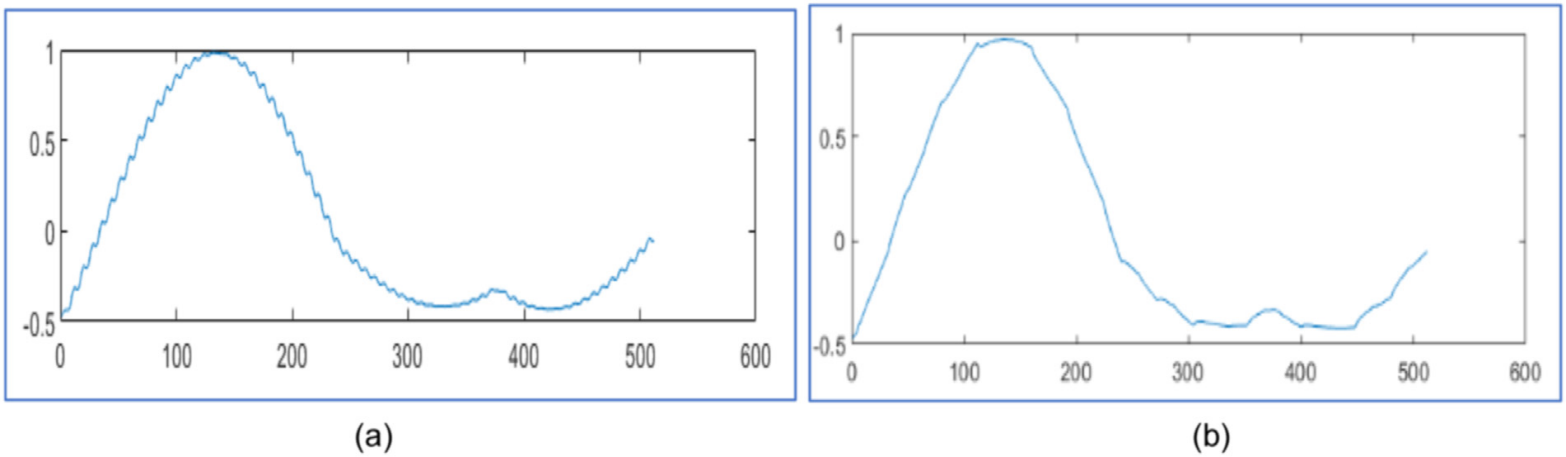
Voltage sag signal reconstruction from 512 samples (a) 99.88 % by proposed design using WPT with 93 samples (b) 99.66 % in [25] with 93 samples.

Fig. 6 represents the original voltage sag signal with 412 samples and the proposed design using WPT reconstructs it 99.97 % as shown in Fig. 7(a) with 81 samples and algorithm in [25] regenerates it 99.91 % with 81 samples as displayed in Fig. 7(b). Similarly Figs. 8, 10, Fig. 12, Fig. 14 represent original voltage sag signals with different samples. Figs. 9(a),11(a), Fig. 13, Fig. 15 are their respective reconstructed signals by proposed design. Figs. 9(b),11(b),Fig. 13, Fig. 15(b) are their respective reconstructed signals by algorithm in [25].

The original voltage sag signal with 357 samples is given in Fig. 8. The proposed design using WPT recreates it 99.97 % with 69 samples, as shown in Fig. 9(a), and with 69 samples, as shown in Fig. 9(b). The algorithm in [25] regenerates it with 99.91 %. The original voltage sag signal is presented in Fig. 10 with 379 samples. The proposed design using WPT regenerates it with 72 samples and 99.98 % accuracy, as shown in Fig. 11(a). The algorithm in [25] regenerates it with 72 samples and 99.79 % accuracy, as shown in Fig. 11(b). The original voltage sag signal with 501 samples is shown in Fig. 12. The proposed design utilizing WPT recreates it 99.90 % with 94 samples, as shown in Fig. 13(a), and with 94 samples, as shown in Fig. 13(b). The approach in [25] regenerates it with 99.33 %. The original voltage sag signal with 512 samples is shown in Fig. 14. The proposed design using WPT reconstructs it 99.88 % from 93 samples, as shown in Fig. 15(a), and the algorithm in [25] regenerates it 99.66 % with 93 samples, as shown in Fig. 15(b). The proposed design regenerates the signal more effectively than [25] utilizing WPT. It compares regenerated signals by proposed design in Table 3 with results in [25] and [28].

Table 3 compares results of voltage sag signal with [25] and [28] and obtains 19.33 % compression, 99.97 % reconstruction and NRMSE 1 of 5.20x$10^{-4}$ by proposed design from 357 original samples. It compresses 19.66 %, reconstructs 99.97 % and NRMSE 1 of 4.69x$10^{-4}$ from 412 original samples. It regenerates 99.98 %, compresses 19.00 %, and has an NRMSE 1 of 4.98x $10^{-4}$ from 379 original samples. It regenerates 99.90 % samples, compresses 18.76 %, and has an NRMSE 1 of 3.69x $10^{-4}$ from 501 original samples. It reduces the signal 19.53 %, regenerates 99.89 % and NRMSE 1 of 2.86 x$10^{-4}$ from 512 original samples. The results are better than [25] and [28]. Compared to [28], the suggested design has produced a higher percentage of the signal being reconstructed. With the same percentage of signal compression, the suggested design has a superior NRMSE 1 than [25].

The algorithms in [25] and [28] are complex as in [25], it uses db3, db2, db2, db2 and db1 wavelets to decompose the signal at level 5 and [28] operates matrices. The proposed design utilizes db3, db2 and db1 with level 3, hence it is less complex as compared to [25] and [28].

## Conclusions and future scope

The proposed design is based on the wavelet packet transform, which compresses and reconstructs data. The proposed design obtained better compression and reconstruction at level 3 with lower order wavelets as per Table 1, Table 2, Table 3. The proposed design has compressed pmu current magnitude signal to 16.15 % with NRMSE 2 of 1.2 x $10^{-3}$ in Table 1. The proposed design has compressed a voltage sag signal with CR 25.46 % and NMSE of 5.14 x $10^{-5}$ in Table 2. Also, Table 3 presents a comparison of the proposed design for various voltage sag signal samples. From 357 original samples, the suggested approach achieves 19.33 % compression and NRMSE 1 of 5.20 x $10^{-4}$. It has an NRMSE 1 of 4.98 x $10^{-4}$ from 379 original samples and compresses 19.00 %. From 412 original samples, it compresses 19.66 % with an NRMSE 1 of 4.69 x$\;10^{-4}$. It has an NRMSE 1 of 3.69x $10^{-4}$ from 501 original samples and compresses 18.76 %. From 512 original samples, it decreases the signal by 19.53 % and the NRMSE 1 of 2.86 x $10^{-4}$. Better signal compression, a higher percentage of the signal being reconstructed, and an improved NRMSE 1, NRMSE 2 and NMSE have all been achieved with the proposed design. Compression and data storage by proposed design are economical, and it sends the data immediately. The present design is computationally not complex as it employs fewer lower-order wavelet filters at level 3. There is no need to decide appropriate subtree. There is flexibility to use different available filters. It has future scope for further improving data compression and reconstruction.

## CRediT authorship contribution statement

**Rakhi Jadhav:** Methodology, Software, Writing – original draft, Data curation, Investigation. **Anurag Mahajan:** Supervision, Validation, Writing – review & editing.

## Declaration of competing interest

The authors declare that they have no known competing financial interests or personal relationships that could have appeared to influence the work reported in this paper.

## Data Availability

Data will be made available on request.
